# Rapidly Progressive Granulomatous Amoebic Encephalitis in a Diabetic Individual

**DOI:** 10.7759/cureus.19336

**Published:** 2021-11-07

**Authors:** Anish C Paudel, Nitin Patel, Jonathan Quang, Courtney Casella, Adam Sigal, Prem Parajuli, Olubunmi Oladunjoye, Ibiyemi O Oke, Suravi Khanal, Kristina Bhattarai

**Affiliations:** 1 Internal Medicine, The Reading Hospital, West Reading, USA; 2 Infectious Disease, The Reading Hospital, West Reading, USA; 3 Emergency Medicine, University of California Irvine Medical Center, Irvine, USA; 4 Emergency Medicine, The Reading Hospital, West Reading, USA; 5 Internal medicine, The Reading Hospital, West Reading, USA; 6 Internal Medicine, Manipal College of Medical Sciences, Pokhara, NPL; 7 Internal Medicine, Nepal Medical College, Kathmandu, NPL

**Keywords:** ring-enhancing lesions, free-living amoeba, amoebic encephalitis, granulomatous amoebic encephalitis, balamuthia mandrillaris

## Abstract

We present a case of rapidly progressive granulomatous amoebic encephalitis caused by *Balamuthia mandrillaris* in an individual with diabetes mellitus. Our patient presented with occipital headache, blurry vision, confusion, and gait imbalance of one week's duration. Brain imaging revealed numerous peripheral ring-enhancing lesions concerning malignancy. Brain biopsy was consistent with *Balamuthia mandrillaris *infection. He passed away 45 days after presentation despite being treated with a five-drug regimen. This case highlights the importance of considering amoebic brain infections, especially with ring-enhancing lesions on imaging. There are opportunities to design modalities for rapid diagnosis and better treatment.

## Introduction

Amoebic encephalitis is a fatal disease with a mortality rate of over 95% [1]. Three major genera of free-living protozoan parasites, namely, *Naegleria fowleri*, *Acanthamoeba*, and *Balamuthia mandrillaris*, have been identified to cause amoebic encephalitis [2]. Since it was first isolated from the brain of a pregnant baboon in 1986, there have been around 200 case reports of *Balamuthia mandrillaris* meningoencephalitis worldwide [1,3]. This rarity of the disease makes the diagnosis challenging and requires high clinical suspicion. Treatment requires a multispecialty approach and guidance from the Centers for Disease Control and Prevention (CDC). A combination drug regimen is often used for its treatment, but the mortality remains high [2]. We present a case of a 51-year-old male with diabetes mellitus not controlled on oral hypoglycemic agents* *who had a rapidly progressive course of *Balamuthia mandrillaris* encephalitis.

## Case presentation

A 51-year-old Hispanic male presented to the hospital following one week of occipital headache, occasional confusion, blurry vision, and gait imbalance. His past medical history was significant for type 2 diabetes mellitus on oral hypoglycemic agents. He initially noted a mild 4/10 occipital headache associated with blurred vision. He denied fevers, loss of consciousness, seizure-like activity, eye pain, photophobia, prior prescription glass use, weight changes, falls, trauma, or rash. The patient migrated from Mexico 22 years ago and had worked as a landscaper ever since. He denied being around recreational water areas and animal exposure to either farm cattle or wild animals. The patient did not drink alcohol or smoke cigarettes. His past medical history and family history were noncontributory.

Upon initial assessment, his vitals were stable. He was fully alert and oriented with clear speech. He reported that his vision was better since he arrived at the hospital. His complete neurological examination was normal.

**Figure 1:**
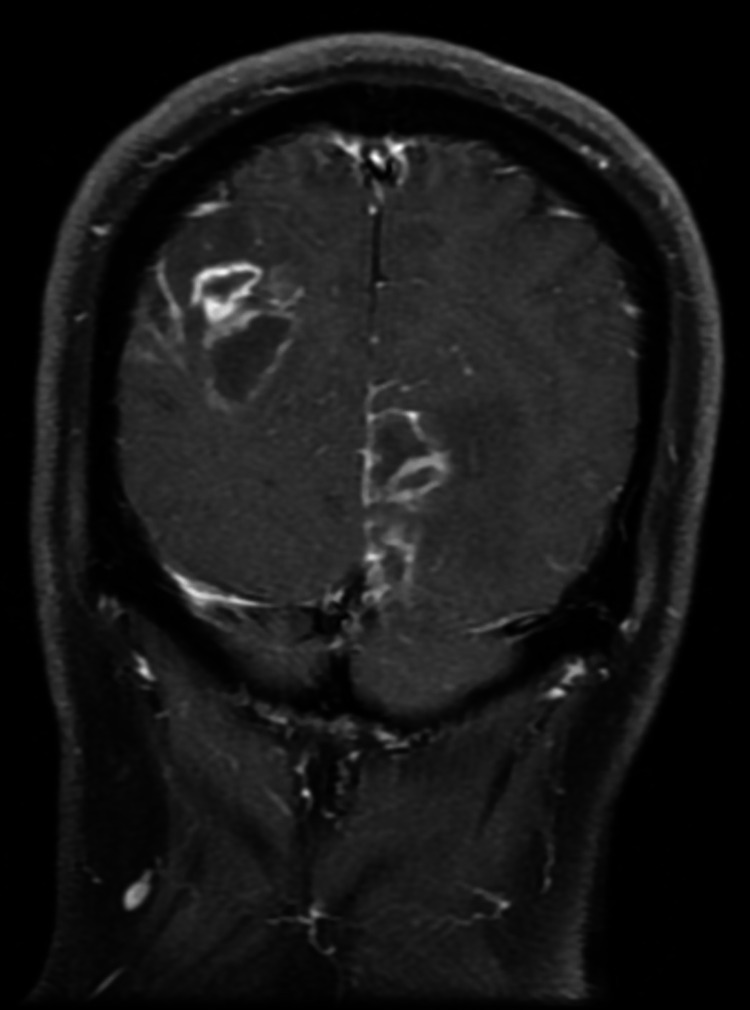
Multiple ring-enhancing lesions noted in a coronal section of brain MRI

The initial set of laboratory tests was positive for an elevated blood glucose level of 175 (normal range: 70-99) mg/dL, hemoglobin A1c of 12.2% (normal range: 4.9%-6%), mildly elevated alkaline phosphatase (ALP) of 125 (normal range: 34-104) IU/L, and lactic acid of 2.4 (normal range: 0.6-1.4) mmol/L. However, ALP and lactic acid normalized without any intervention. His white blood cell (WBC) count and differentials were within normal limits. Non-contrast CT scan of the brain revealed multiple foci of decreased attenuation throughout both cerebral hemispheres with preservation of grey-white differentiation. A 3 mm sulcal-based calcification was noted within the right frontal lobe. The findings were suggestive of multifocal intracranial lesions related to metastasis or less likely infection. MRI revealed numerous peripheral enhancing lesions throughout the brain, demonstrating central liquefaction and surrounding edema, as well as possible components of petechial hemorrhage (Figure 1). One lesion in the right occipital lobe measured 19 mm. Other lesions were present in the left occipital region, right frontal-parietal region, left cerebellum, and left temporal lobe. Additionally, smaller lesions were scattered elsewhere throughout the brain. Differential included cerebral abscess and metastasis.

CT scan of the chest, abdomen, and pelvis was ordered with concerns for metastatic evaluation but was unrevealing. The patient received one dose of 4 mg intravenous dexamethasone, which was not continued. CSF serologies were negative for HIV and *Toxoplasma gondii*. The patient underwent a biopsy of the right frontal lobe lesion. A day after the biopsy, the patient complained of sudden left arm weakness, left-sided facial droop, and slurred speech. Strength testing was significant for 4/5 left upper extremity weakness. Repeat non-contrast CT scan of the head showed a 37 mL intracranial hemorrhage with mild intravascular extension. No intervention was done as repeat CT scans were stable.

**Figure 2:**
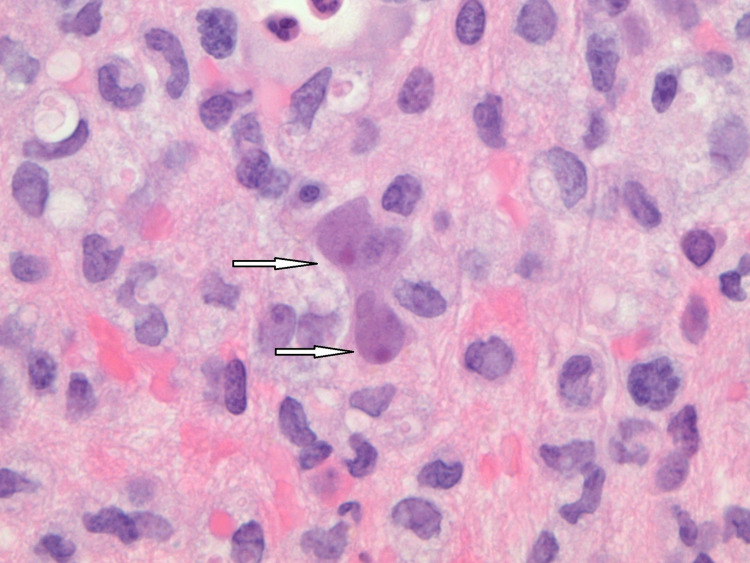
Trophozoites of Balamuthia mandrillaris (arrows) in hematoxylin and eosin stain under oil immersion

Cultures from the brain biopsy samples were negative for any growth, including acid-fast bacilli. Histopathology revealed multiple amoebic forms with basophilic cytoplasm, large nuclei, and prominent nucleoli with predominant perivascular arrangements (Figure 2). These morphological findings were consistent with *Balamuthia mandrillaris*.

He was started on a combination regimen of flucytosine, fluconazole, azithromycin, sulfadiazine, and miltefosine. Pentamidine was not added due to concerns of toxicity. The pathologic slides were sent to CDC for PCR analysis, but unfortunately, no significant specimen was left to do the test. The patient was subsequently discharged to an acute rehabilitation hospital continuing on the same regimen. During his two weeks' stay at the rehabilitation hospital, he had intermittent hallucinations and was found to have decreased cognition. He was brought to the neurosurgery clinic six days after discharge from the rehabilitation hospital with increasing somnolence and worsening confusion. Repeat CT scan showed hydrocephalus, for which he underwent an external ventricular drain placement. A CSF specimen was sent to the CDC for PCR analysis that was positive for *Balamuthia mandrillaris* species. Unfortunately, the patient had worsening encephalopathy and developed respiratory distress. The patient was placed on hospice, where he passed away.

## Discussion

Free-living amoeba exists ubiquitously in natural environments and, as their name suggests, do so without the need for a definitive host. They are well documented to cause pathogenic infections primarily of the CNS. Among the well-known amoebic species, *Acanthamoeba* and *Balamuthia mandrillaris *cause granulomatous encephalitis in immunocompromised or debilitated individuals, whereas *Naegleria fowleri* causes necrotizing and hemorrhagic fulminant meningoencephalitis in children and young adults [2]. Although infections are rare, they tend to be consistently fatal even with antimicrobial therapies. Therefore, early diagnosis and initiation of treatment represent an essential means to mitigate the lethality of the disease process. The case presented in this report features some crucial nuances to the diagnosis and management of granulomatous amoebic encephalitis caused by *Balamuthia mandrillaris*.

The rarity of granulomatous amoebic encephalitis poses a diagnostic challenge and demands a high degree of clinical suspicion. Identifying risk factors and exposures are therefore crucial to early diagnosis. *Balamuthia mandrillaris *specifically has been reported to be found in soil, dust, and water [4]. In one of the largest case series published on *Balamuthia mandrillaris*, soil exposure history was present in 85% of cases where it was documented [5]. Swimming or recreational water exposure was reported in 66% of cases. The case series also identified two other demographic patterns. Males represented 68% of cases, and Hispanics represented 55% of cases where ethnicity was reported. The authors noted that these patterns might reflect demographics for occupations with heavy soil exposure, such as agriculture or landscaping. The patient in this case report was notably 1) a landscaper and 2) of Hispanic ethnicity. Interestingly, most cases also occurred in immunocompetent individuals. Immunocompromisation was reported in less than 40% of cases.

Unfortunately, the presenting symptoms for *Balamuthia mandrillaris *are primarily nonspecific. The patient presented above reported a mild headache, blurred vision, and gait imbalance. The vague, nonspecific, and often mild symptomatology in early disease obfuscate diagnosis and any decisions for imaging. It would be easy to dismiss a rare diagnosis for a much more common one, such as migraine or viral syndrome. The case series mentioned above supports this notion. The most common clinical features on presentation were noted to be fever (39%), headache (39%), vomiting (30%), and lethargy (28%) [5]. In the case presented, the decision to pursue imaging was largely gestalt, emphasizing the importance of considering a broad differential diagnosis and neuroimaging in headaches atypical to the patient’s baseline.

Cutaneous findings have been postulated to precede CNS infection by weeks or months [6], representing an opportunity for early diagnosis and intervention. The rash has been described as an asymptomatic plaque (single or with satellite lesions) with a poorly defined border, most commonly located on the central face or in one extremity [6]. Cutaneous findings overall are variable. While a series of 55 cases in Peru [6] found most of their patients presented with cutaneous findings, the only other large-case series [5] found only 5% of their patients to have cutaneous lesions. The patient in this case report had no cutaneous findings, which could also suggest if there was a direct transmission of the pathogen through the nasopharynx*. *Nonetheless, examination of the skin should remain part of a complete assessment as it provides a potential opportunity to halt disease progression to the CNS, where it becomes universally fatal.

The initial non-contrast CT imaging of the head in our patient revealed numerous foci of decreased attenuation. It was the first distinguishing finding that narrowed the differential and warranted focused investigation. Subsequent contrast MRI showed numerous ring-enhancing lesions. Again, a previous case series found that all cases had abnormal CT and MRI with enhancing lesions (29%), multifocal lesions (23%) being among the most common findings [5]. There did not appear to be any regional preference for infection.

General CSF studies in our patient did not help differentiate the amoebic causes of meningoencephalitis from other causes. In general, patients tend to have mildly elevated WBC and protein with normal levels of glucose. The gold standard for diagnosis is biopsy with indirect immunofluorescent staining of brain tissue sections. Real-time PCR may provide a more rapid means (five hours) of identifying *Balamuthia mandrillaris *in either brain tissue or from the much more accessible CSF. These tests, however, are only available at the Centers for Disease Control and Prevention in Atlanta, Georgia. When amoebic meningoencephalitis is first suspected, early involvement with the CDC is crucial as they provide valuable guidance and expedite the testing process.

Limited evidence exists on the treatment of granulomatous amoebic encephalitis caused by *Balamuthia mandrillaris*. Previous case reports and in vitro data primarily guide antimicrobial therapy. The CDC currently recommends a combination of flucytosine, pentamidine, fluconazole, sulfadiazine, and either azithromycin or clarithromycin [7]. There has also been emerging evidence supporting the use of miltefosine [8]. Despite the use of these therapies, mortality remains high. More research is needed to find the optimal treatment regimen.

*Balamuthia mandrillaris *and, more broadly, amoebic CNS infections present unique diagnostic and therapeutic challenges. Early multispecialty support and involvement with the CDC are critical to expeditious diagnosis and initiation of treatment. Delays in diagnosis and lack of evidence for treatment likely represent the biggest impediments to reducing mortality. Although the prognosis is poor, case reports of survivors and promising evidence for new drugs are hopeful. Further case reports, research, and trials are needed.

## Conclusions

*Balamuthia mandrillaris* is a free-living protozoan parasite that can cause amoebic encephalitis often in an immunocompromised host. This infection can progress rapidly and causes fatal necrotizing and hemorrhagic fulminant meningoencephalitis. Its rarity demands a high degree of clinical suspicion for timely diagnosis. There are no standardized treatment options to treat these infections. We will need more research and data to come up with a good treatment regimen.
